# Free-Standing and Self-Crosslinkable Hybrid Films by Core–Shell Particle Design and Processing

**DOI:** 10.3390/nano7110390

**Published:** 2017-11-15

**Authors:** Steffen Vowinkel, Stephen Paul, Torsten Gutmann, Markus Gallei

**Affiliations:** 1 Ernst-Berl-Institute for Chemical Engineering and Macromolecular Science, Technische Universität Darmstadt, Alarich-Weiss-Str. 4, D-64287 Darmstadt, Germany; s.vowinkel@mc.tu-darmstadt.de; 2 Eduard-Zintl Institute for Inorganic and Physical Chemistry, Technische Universität Darmstadt, Alarich-Weiss-Str. 8, D-64287 Darmstadt, Germany; Stephen-paul@t-online.de (S.P.); gutmann@chemie.tu-darmstadt.de (T.G.)

**Keywords:** hybrid films, colloidal crystals, self-assembly, colloids, particle processing, emulsion polymerization, cross-linking

## Abstract

The utilization and preparation of functional hybrid films for optical sensing applications and membranes is of utmost importance. In this work, we report the convenient and scalable preparation of self-crosslinking particle-based films derived by directed self-assembly of alkoxysilane-based cross-linkers as part of a core-shell particle architecture. The synthesis of well-designed monodisperse core-shell particles by emulsion polymerization is the basic prerequisite for subsequent particle processing via the melt-shear organization technique. In more detail, the core particles consist of polystyrene (PS) or poly(methyl methacrylate) (PMMA), while the comparably soft particle shell consists of poly(ethyl acrylate) (PEA) and different alkoxysilane-based poly(methacrylate)s. For hybrid film formation and convenient self-cross-linking, different alkyl groups at the siloxane moieties were investigated in detail by solid-state Magic-Angle Spinning Nuclear Magnetic Resonance (MAS, NMR) spectroscopy revealing different crosslinking capabilities, which strongly influence the properties of the core or shell particle films with respect to transparency and iridescent reflection colors. Furthermore, solid-state NMR spectroscopy and investigation of the thermal properties by differential scanning calorimetry (DSC) measurements allow for insights into the cross-linking capabilities prior to and after synthesis, as well as after the thermally and pressure-induced processing steps. Subsequently, free-standing and self-crosslinked particle-based films featuring excellent particle order are obtained by application of the melt-shear organization technique, as shown by microscopy (TEM, SEM).

## 1. Introduction

Artificial functional materials derived by self-assembly of tailored particles both reveal fundamental insights into colloidal crystallization and access to organic, inorganic, or hybrid colloidal films. Such highly interesting materials can feature fascinating optical, electrical, or magnetic properties [1,2,3,4,5]. In general, colloidal crystals can be easily obtained by inexpensive and convenient bottom-up approaches leading to materials with sufficient optical performance, i.e., iridescent reflection colors caused by Bragg diffraction of visible light [6,7,8]. Moreover, such artificially generated colloidal crystals can be equipped with so-called smart functionalities, i.e., stimuli-responsive polymers, leading to reversibly remote-switchable polymer opals for a manifold of optical sensing applications. Recent reviews within the field of switchable opal structures are given by, e.g., Ge et al. [9] or other authors [10,11,12,13]. In general, the feasibility of hierarchically structured hybrid materials led to the combination of a plethora of properties within this field [14,15,16]. Hierarchical colloidal architectures with tailored domain sizes have attracted attention because of their tremendous potential for applications in fields of catalysis, separation, sensors, optics, and biomedicine [17,18,19,20]. One major strategy for the preparation of hybrid particle-based films with ordered structures is polymer templating, which is also referred to as soft templating. So-called polymer opals consist of rather monodisperse submicroscopic particles arranged in a face-centered cubic (fcc) lattice. The particle order can be accomplished in a simple fashion by various techniques of drying, deposition, or by spin coating from the particle dispersions [5,6]. However, the scalable preparation of crack- and defect-free films still remains challenging, as evidenced by the works of Aizenberg et al. [21,22], and the preparation typically involves a multi-step procedure comprising colloidal crystallization, monomer infiltration, and subsequent cross-linking steps [5,6,12]. An alternative approach for particle-based film formation is the so-called melt-shear organization technique [23,24,25]. By applying moderate pressure to the tacky mass of hard core–soft shell particles and while increasing the temperature, the hard core particles can merge into the colloidal crystal structure inside a continuous matrix of soft shell material in one step. Free-standing polymer opals with iridescent reflection colors can thus be produced. Moreover, for the melt-shear organization, no dispersion medium or solvent for the particles is necessary. In the recent past, this technique has been advantageously applied for the large-scale production of polymer opal films and subsequent particle assembly by bending-induced oscillatory shearing (BIOS) [26]. However, a subsequent cross-linking step is necessary in order to obtain stable opal films due to the following reasons: It has to be mentioned that the uncured particle film—free of any cross-linking sites—is soft as a rubber and behaves like a viscous liquid and therefore lacks robustness [27]. For further mechanical improvements, subsequent cross-linking processes were applied after the melt-shear organization of the core-shell particle films for stabilizing the matrix materials of the particle films. Exemplarily, Viel et al. reported on a UV-induced cross-linking strategy by using benzophenone as radical photo-cross-linker [28]. After this additional step, the final elastomeric opal films could be strained, and the opal film deformation proved to be almost reversible for mechanochromic sensing applications. Schäfer et al. developed a thermally induced cross-linking route as subsequent step after the melt-shear organization process, which proved to be feasible for thicker particle films (above 200 µm) in order to generate a homogeneous cross-linking density [23]. Additionally, the melt-shear organization technique was used for the preparation of opal films consisting of inorganic particles and soft polymer shell materials [29,30,31]. In order to avoid additional cross-linking steps after particle processing and to overcome issues with cross-linking efficiencies for thicker films (>150 μm), self-cross-linking reactions should be taken into consideration.

In the present study, three different siloxane-containing methacrylate monomers, i.e., 3-methacryloxypropyltriethoxysilane (MPSEt), 3-methacryloxypropyltriisopropoxysilane (MPSIsoprop), and 3-methacryloxypropyltris(methoxyethoxy)silane (MPSMeEt) are studied as soft-shell polymers for hybrid film formation by application of the melt-shear organization technique. The cross-linking kinetics and hybrid film formation are elucidated by investigating thermal properties of the core-shell particles, variation of the processing parameters followed by scanning electron microscopy (SEM) of the obtained films, and by solid-state NMR spectroscopy. The insights into the cross-linking kinetics and comparison of the different hybrid particle architectures offer the possibility to produce hierarchically ordered hybrid film materials in one processing step without use of further subsequent cross-linking strategies.

## 2. Experimentation

### 2.1. Materials

Methyl methacrylate (MMA), allyl methacrylate (ALMA) and styrene were obtained from Fisher Scientific (Schwerte, Germany), ethyl acrylate (EA) from BASF SE (Ludwigshafen, Germany), and Dowfax 2A1 from Dow Chemicals (Midland, MI, USA). The siloxane-containing methacrylates were purchased from ABCR (Karlsruhe, Germany). All other chemicals were obtained from Sigma-Aldrich (Munich, Germany). The inhibitors were removed from the monomers by passing through a basic alumina column. All other substances were used as received.

### 2.2. Synthesis of PMMA-co-ALMA and PS-co-ALMA Core Particles

The core particles were synthesized in a 1 L double-wall reactor equipped with a stirrer and reflux condenser under argon atmosphere. A dispersion of 440 g water, 3.6 g MMA, 0.4 g ALMA, and 50 mg sodium dodecylsulfate (SDS) was filled into the reactor for seed particles synthesis. For PS particles, MMA was replaced by styrene and the amount of SDS was adjusted to 250 mg (300 mg SDS for smaller-sized PS cores). The emulsion polymerization was initiated by the addition of 50 mg sodium bisulfate, 150 mg sodium persulfate for PMMA particles synthesis (500 mg for PS particle synthesis), and 50 mg sodium bisulfate, each dissolved in 5 mL of water and added in this order. After 15 min, the monomer emulsion consisting of 170 mg SDS, 100 mg KOH, 120 mg Dowfax 2A1 72 g water, 50.4 g MMA, and 5.6 g ALMA was continuously added with a speed of 1.2 mL min^−1^ using a rotary piston pump. For PS particle synthesis, the monomer emulsion consisted of 230 mg SDS, 200 mg KOH, 230 mg Dowfax 2A1, 90 g water, 70 g styrene, 7 g ALMA, and was continuously added with a speed of 1 mL min^−1^ utilizing a rotary piston pump Reglo-CPF digital, RH00 (Ismatec, Wertheim, Germany).

### 2.3. Particle Shell Formation by Copoylmerization of Alkoxysilane-Containing Methacrylates with Ethyl Acrylate

Three different alkoxysilane-containing methacrylates were used for particle shell synthesis for the PMMA core particles. For this purpose, in a 250 mL double-wall reactor equipped with a stirrer and reflux condenser under argon atmosphere, the core particle dispersion was added and buffered with 4 mL phosphate buffer (0.1 M) to pH 7. As initiator, 150 mg sodium persulfate was added. After 15 min, the monomer emulsion consisting of 30 mg SDS, 30 mg Dowfax 2A1, 5.82 g water, 180 mg phosphate buffer (0.1 M), and 5.15 g of the respective alkoxy-silane monomers were continuously added with a speed of 0.27 mL min^−1^ utilizing a rotary piston pump. As monomers, ethyl acrylate with 15 mol % of the alkoxysilane-containing methacrylates 3-methacryloxypropyltriethoxysilane **1** (MPSEt), 3-methacryloxypropyl-triisopropoxysilane **2** (MPSIsoprop), and 3-methacryloxypropyltris(methoxyethoxy)silane **3** (MPSMeEt) were used.

For the shell synthesis of the two different PS particle batches, the procedure was nearly identical as for the shell synthesis of the PMMA particles. Only the added monomer emulsion was modified, which consisted of 45 mg SDS, 45 mg Dowfax 2A1, 6 g water, 4.79 g ethyl acrylate, 0.53 g MPSEt, and 180 mg phosphate buffer (0.1 M). The ratio of cross-linker to monomer correlates to 3.7 mol %.

### 2.4. Melt-Shear Organization Procedure

For the preparation of the opal film, the hybrid core-shell particle mass was lyophilized and 750 mg of the obtained powder was enclosed between two polyethylene terephthalate (PET) foils. The powder between the foils was inserted into a Collin laboratory press P200 P/M (Dr. Collin GmbH, Ebersberg, Germany) and the particle films were prepared by using the melt-shear organization technique at different temperatures, ranging from 115 °C to 140 °C, and pressures ranging from 140 bar to 180 bar for 3 min.

### 2.5. Characterization

Transmission electron microscopy (TEM) measurements were carried out on a Zeiss EM109 (Oberkochen, Germany) electron microscope operating at 80 kV. The shown images were obtained with a Gatan BioScan camera (Gatan, Pleasanton, CA, USA). For dynamic light scattering (DLS) measurements, a Zetasizer ZS90 (Malvern Instruments, Malvern, UK) was used. Differential scanning calorimetry (DSC) was performed with a Mettler Toledo DSC-1 (Columbus, OH, USA) with a heating rate of 5 K min^−1^. For scanning electron microscopy (SEM), a FEI/Philips XL30 FEG (Hillsboro, OR, USA) with accelerating voltages between 5 and 20 kV was used. The SEM samples were coated with gold for 100 s at 30 mA using a Quorum Q300T D (Laughton, UK) sputter coater. For UV/Vis measurements, a vis-NIR fiber spectrophotometer USB 4000 (Ocean Optics, Dunedin, FL, USA) with a deuterium/tungsten halogen lamp (HL-2000, Ocean Optics, Dunedin, FL, USA) was used. All solid-state NMR spectra were measured on a Bruker Avance III HD 600 spectrometer (Rheinstetten, Germany) employing a 4 mm broad band H/X probe. Spectra were recorded at 14 T, corresponding to a frequency of 119.23 MHz for ^29^Si and 150.90 MHz for ^13^C, at 8 kHz and 10 kHz spinning, respectively, at room temperature. Spectra were referenced to tetramethylsilane (TMS), employing kaolinite (−92.5 ppm) for ^29^Si and adamantane (+38.5 ppm) for ^13^C as external standards. The ramped CP-MAS sequence [32] was utilized with a contact time of 3.5 ms for ^29^Si and 5 ms for ^13^C. The recycle delay was set to 4 s and tppm decoupling [33] employing a 15° phase jump, was applied during data acquisition. ^29^Si and ^13^C static measurements were performed employing single pulse excitation with a 30° pulse and a recycle delay of 4 s.

## 3. Results and Discussion

### 3.1. Synthesis Hybrid Particles with Alkoxysilane-Containing Methacrylates

**Figure 1:**
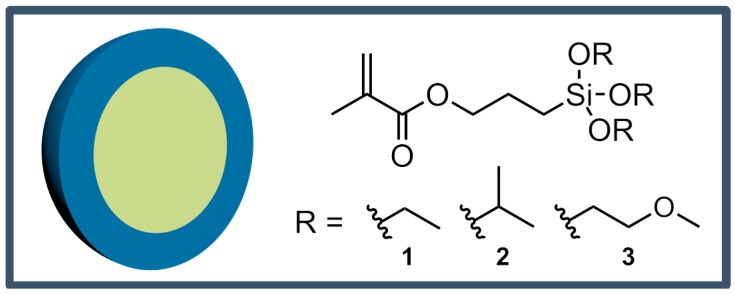
Particle architecture consisting of either P(MMA-*co*-ALMA) or P(S-*co*-ALMA) cores and a shell of poly(ethyl acrylate) with different alkoxysilanes as comonomers, i.e., MPSEt **1**, MPSIsoprop **2**, MPSMeEt **3**.

Starved-feed emulsion polymerization was carried out for the preparation of monodisperse hybrid core–shell particles. The chemical structure of the particles is given in Figure 1: poly(methyl methacrylate) (PMMA) or polystyrene (PS) core particles having a comparably soft shell material of copolymerized ethyl acrylate (EA) with different alkoxysilane-containing methacrylates, i.e., either 3-methacryloxypropyltriethoxysilane **1** (MPSEt), 3-methacryloxypropyltriisopropoxysilane **2** (MPSIsoprop), or 3-methacryloxypropyltris(methoxyethoxy)silane **3** (MPSMeEt).

**Figure 2:**
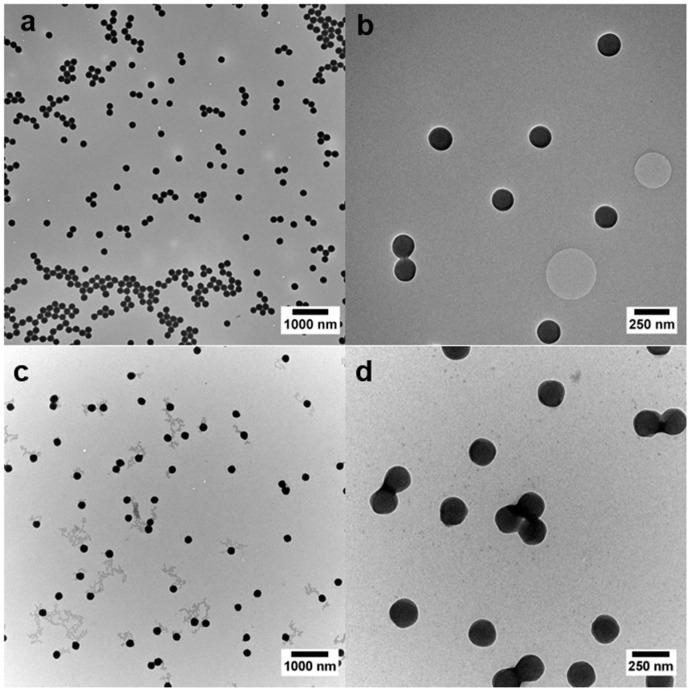
TEM Images of the P(MMA-*co*-ALMA) core particles (**a**,**b**) and the corresponding P(MMA-*co*-ALMA)@P(EA-*co*-MPSEt) core–shell particles (**c**,**d**). Scale bars correspond to 1000 nm (**left**) and 250 nm (**right**), respectively.

Starting from the same core particle batch of PMMA, the hybrid shell was prepared with an amount of 15 mol % of the respective alkoxysilane-containing methacrylate with EA. The calculated amount of monomers (EA and alkoxysilane-methacrylate **1**–**3**) was the same in order to maintain the ratio between core and shell material. The adjustment of the core to shell ratio is one critical parameter for the intended melt-shear organization: too little shell material will lead to a rather hard material that is not capable of merging into the colloidal crystal structure during processing. A large amount of shell material enables better flowing and thus processing ability of the particles, but it will cause less order because of gradation of the particle stacks [26,29,34]. Therefore, an exact control over the particle architecture is mandatory for the melt-shear organization. In order to determine the core–shell ratio and the monodispersity of the particles, transmission electron microscopy (TEM) and dynamic light scattering experiments (DLS) were carried out for each particle synthesis step. Exemplarily, TEM images of the P(MMA-*co*-ALMA) core particles and P(MMA-*co*-ALMA)@P(EA-*co*-MPSEt) are given as Figure 2, while TEM images of all other samples are given as Figures S1–S4 in the Supporting Information.

**Figure 3:**
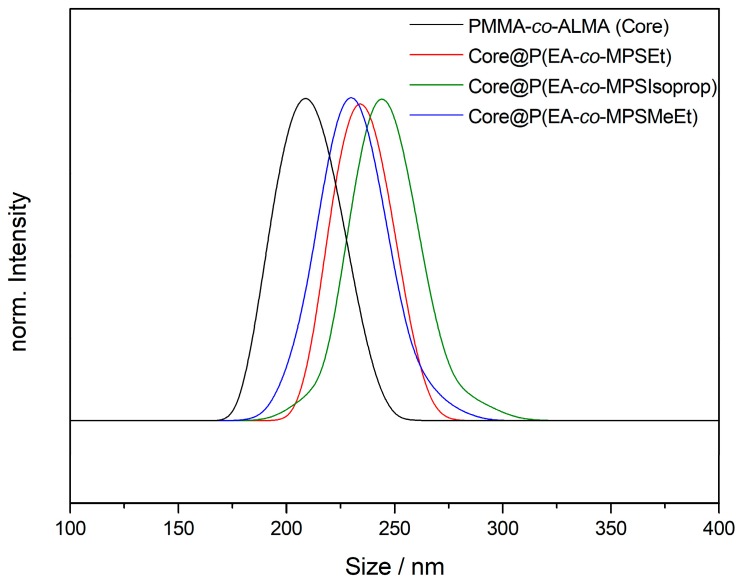
DLS measurement of the PMMA-*co*-ALMA core particles and subsequent steps of the core–shell particle synthesis with different poly(alkoxysilane methacrylate) copolymers with PEA as shell material.

The TEM images in Figure 2 for PMMA-based particles (and Figures S1–S4 for PS-based particles) prove the excellent control over the applied emulsion polymerization for the preparation of monodisperse particles. A crucial aspect during emulsion polymerization was the control over the pH value of the dispersion during the radical polymerization in order to avoid a self-crosslinking reaction of the reactive siloxane moieties. For this purpose, a phosphate buffer was used during polymerization. Another critical point to be mentioned is the applied drying protocol for the obtained particle mass after agglomeration or precipitation: in first attempts, moderate thermal treatment for drying the particle mass in an oven led—again—to cross-linking reactions, impeding the processing capabilities of the particle mass. Therefore, lyophilization of the particle dispersion after synthesis was carried out. Additionally to TEM investigations, the particle size was determined by DLS measurements (Figure 3).

The average particle sizes were determined to be 209 ± 14 nm for the core particles and 234 ± 13 nm for the MPSEt containing core–shell particles, as well as 244 ± 16 nm (MPSIsoprop) and 230 ± 16 nm (MPSMeEt), respectively (additional DLS measurements for PS-based particles in Figure S5). In summary, from the results derived by TEM and DLS measurements, it can be concluded that the particle sizes were similar and hence comparable to each other. This will allow for direct comparison of the different alkoxy-silane methacrylates used with respect to processing and hybrid film formation, which will be described in detail in the following sections.

### 3.2. Investigation of the Cross-Linking Capabilities of the Poly(alkoxysilane-methacrylate)s

**Figure 4:**
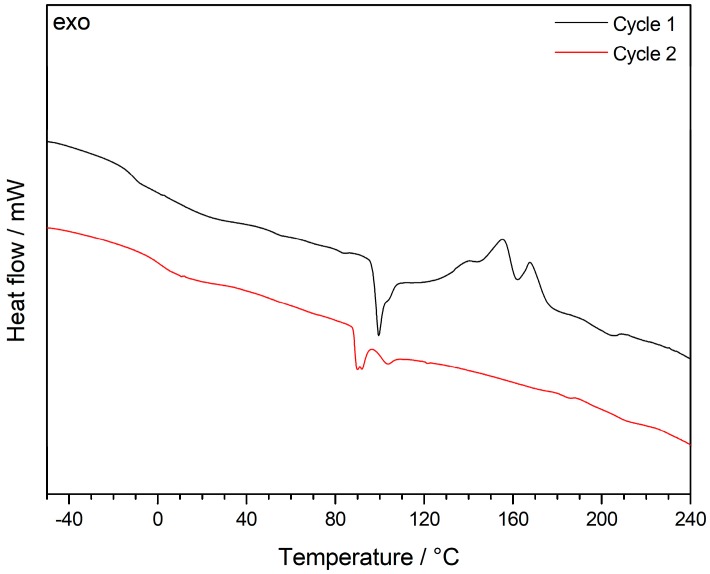
Differential Scanning Calorimetry (DSC) thermograms of PS-*co*-PALMA@P(EA-*co*-MPSEt) lyophilized particle powder with a heating rate of 5 K min^−1^ under nitrogen atmosphere.

As both the features of the core and shell material and the cross-linking behavior of the different alkoxysilane-methacrylates are very important for processing of the particles by melt-shear organization, differential scanning calorimetry (DSC) measurements were carried out. Besides the determination of the corresponding glass transition temperatures, *T*_g_, exothermic cross-linking reactions could be elucidated. At higher temperatures, however, the incipient degradation signals of the PMMA core overlapped with the exothermic cross-linking reaction, as shown in the corresponding thermogram (Figure S6). In order to further study the heat of cross-linking reaction, PS particles as core material were used for DSC measurements in order to unravel the cross-linking reactions of the hybrid shell materials. The intrinsically higher thermal stability of the decorated PS particle system as well as the heat of cross-linking were investigated in the DSC thermogram in Figure 4.

Comparing the first (black) and the second (red) cycle of DSC measurements for the lyophilized powder revealed significant exothermic signals for the first run, which can be assigned to the cross-linking reaction. It is worthy to mention that the cross-linking reaction of the siloxanes seemed to be already complete after the first DSC measurement, as there were no exothermic peaks for the second DSC run. The amount of siloxane-based cross-linker for the PS-core model system was 3.7 mol %. Therefore, a shift of the *T*_g_ before and after thermal cross-linking reaction could be observed, i.e., from −12 °C to 5 °C. This finding is within the expectation for the still soft, but after thermal treatment slightly cross-linked matrix material. Compared to this, the PMMA core particle film feature a high amount of siloxane cross-linkers of 15 mol %. Therefore, the *T*_g_ of the siloxane-containing segments completely disappeared after thermal treatmenst up to 240 °C due to the loss of segmental dynamics for the matrix material (Figure S6). One additional important insight into the system was that only the lyophilization of the particle dispersion provided a thermally cross-linkable material. For DSC measurements of oven-tried particle samples (12 h, 40 °C), no exothermic peaks could be observed in the corresponding thermograms (not shown). As a result, the lyophilization step as method itself is therefore capable of efficiently inhibiting such siloxane crosslinking reactions.

**Figure 5:**
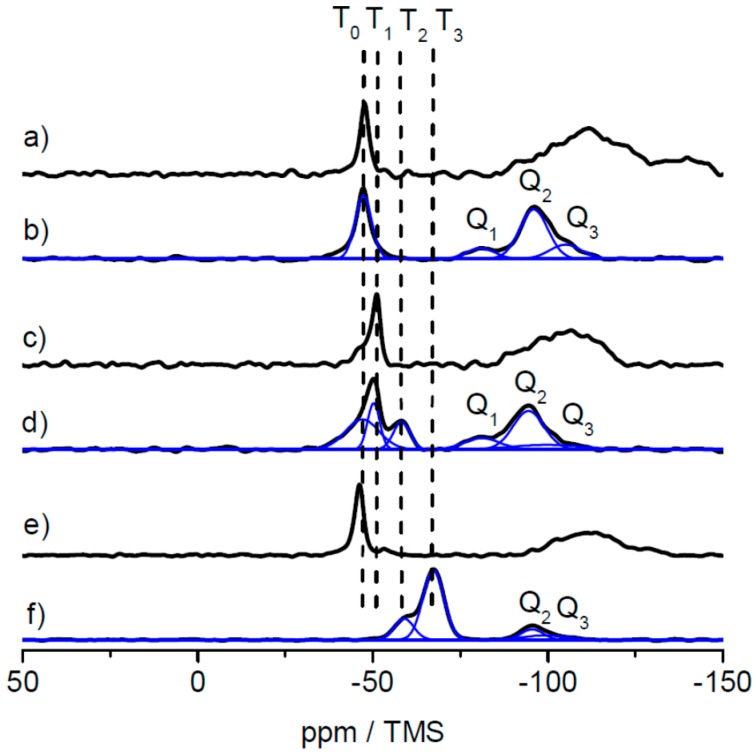
^29^Si static spectra of the free linkers MPSEt (**a**), MPSIsoprop (**c**) and MPSMeEt (**e**), and corresponding ^29^Si CP MAS spectra of the lyophilized powder samples prepared with these linkers (**b**,**d**,**f**). Note: the broad signal in the high field region of the static spectra refers to the rotor insert that was employed to handle the liquid linkers for static measurements with the solid-state NMR probe. The spectra of the lyophilized powder samples were deconvoluted to ease the signal assignment to different structural elements.

In order to investigate the three different alkoxysilane-methacrylates MPSEt, MPSIsoprop, and MPSMeEt cross-linked siloxane sites after lyophilization, solid-state NMR measurements were performed. While TEM and DLS measurements allow for the identification of the shape and the average size of the core and core–shell particles, the analysis of the cross-linking sites of siloxane moieties necessitates a characterization at the molecular level. In principle, vibrational spectroscopy techniques such as IR and Raman spectroscopy allow such analysis. However, these techniques suffer from resolution, which makes their interpretation challenging, especially when complex hybrid material containing inorganic and organic components are involved. As an alternative to solve this structural puzzle, solid-state NMR has been evaluated to characterize inorganic-organic hybrid materials, and the interactions of organic components with inorganic ones [35,36,37,38,39,40,41,42,43,44,45]. This technique has the advantage that the nuclei of the inorganic component, i.e., ^29^Si, and the nuclei of the organic component, i.e., ^13^C, have specific chemical shift ranges that allow a precise analysis of their chemical environment and thus the clear identification of functional groups and their connectivity. Many examples of silica hybrid materials containing organic polymers are found in the literature [30,46,47,48,49,50,51,52], where the potential of ^29^Si and ^13^C solid-state NMR techniques were demonstrated to obtain detailed structural information. Figure 5 shows the ^29^Si static spectra of the three linker systems and the corresponding ^29^Si CP MAS spectra of the lyophilized powder samples for comparison. All ^29^Si CP MAS spectra show typical signals in the range of *T_n_* groups (*n* = 0, 1, 2, 3) (between ca. −40 and −75 ppm) where *n* represents the number of –O–Si– bonds at the silicon atom of the linker molecule. This number *n* is a measure for the degree of cross-linking via silicon of the linker molecules [53,54]. With increasing *n*, the cross-linking character via silica increases. Possible structures for the MPSEt linker system assigning *T_n_* groups are shown in Figure S7. Although ^29^Si CP MAS NMR is not quantitative, the progress of cross-linking can be approximated by analyzing the spectra line shape. Comparing the *T_n_* group patterns for the different linker systems in Figure 5, it is clearly visible that in the lyophilization step the cross-linking degree varies in the order MPSEt < MPSIsoprop < MPSMeEt. This means that MPSMeEt has the highest probability to cross-link in the lyophilization step.

Interestingly, the ^29^Si CP MAS spectra also show signals in the range of *Q_n_* groups (*n* = 1, 2, 3) (between ca. −80 and −105 ppm) [55] illustrated in Figure S7 which refer to “bulk silica”—that is, derived from Si(OH)_4_ by cross-linking. The origin of this “bulk silica” is not completely clear but it can be assumed that its formation refers to side products that appeared in the synthesis of the linker systems. For the sake of completeness, the comparison of ^13^C static spectra of the three linker systems and the corresponding ^13^C CP MAS spectra of the lyophilized powder samples are shown in Figures S8–S10. These spectra in principle provide information on the linking moieties of the organic component. Comparison of the three linker systems reveals no significant differences in the line shape of the ^13^C CP MAS spectra obtained for the lyophilized powder samples. This is not very surprising, since these spectra are dominated by ^13^C signals referring to the polymer core of the particles. According to the results from DLS measurements (see above) the diameter of the shell is only 10% of the diameter of the particles. Thus, linker-dependent differences of the shell structure of the particles are not clearly visible in the ^13^C CP MAS spectra.

### 3.3. Hybrid Film Formation by Melt-Shear Organization

**Figure 6:**
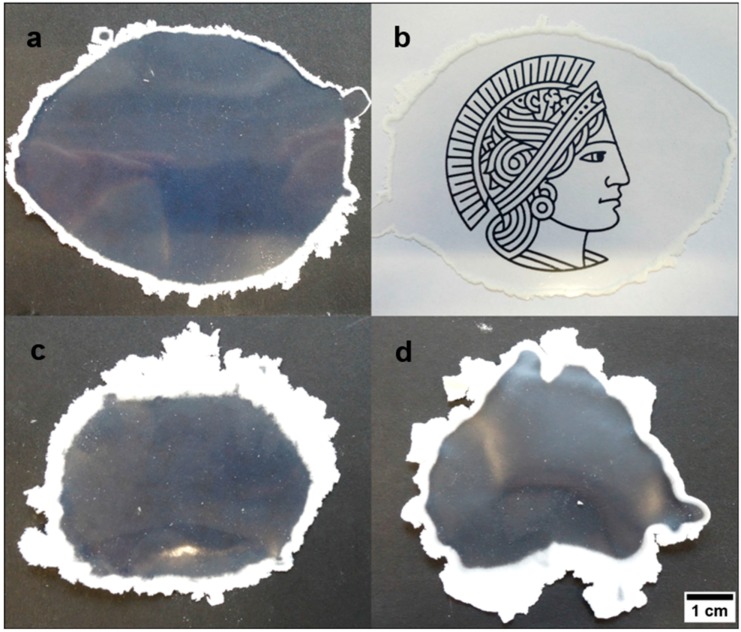
Photos for the particle-based films obtained after melt-shear organization at 140 °C, 180 bar, for 180 s. In detail, the core–shell particles consisted of a P(MMA-*co*-ALMA) core and a shell material consisting of the investigated siloxane-containing polymers, i.e., P(EA-*co*-MPSEt) (**a**,**b**), P(EA-*co*-MPSIsoprop) (**c**), and P(EA-*co*-MPSMeEt) (**d**). The scale bar corresponds to 1 cm.

As concluded from the results in the previous section, the particle dispersion obtained after emulsion polymerization had to be lyophilized for the ensuing processing steps. The obtained particle powder was subjected to the melt-shear organization. For this purpose, the particle powder was transferred between the plates of a moderately hot press sandwiched with a PET foil (see Experimentation Section). The optimized processing conditions to obtain homogeneous hybrid particle films were determined to be 140 °C processing temperature and 180 bar pressure for 180 s. In the case of P(MMA-*co*-ALMA) core particles, the particle films feature a high transparency because of the low refractive index (cf. *n*_PMMA_ = 1.4906; *n*_PEA_ = 1.4685) contrast between the core and the hybrid poly(methacrylate) shell material. The influence of the different siloxane-containing poly(methacrylate) copolymers with PEA were investigated with respect to their optical features (transparency, size of molten transparent film). The obtained particle films having the three different siloxane-polymethacrylates incorporated in the shell after melt-shear organization are displayed in Figure 6.

From the optical impressions in Figure 6, the quality of the obtained particle films can be evaluated by comparing the transparency of the inner area of the film and the width of the white areas surrounding the film. The white particle powder surrounding the transparent inner film area indicated the incapacity of particle merging during the melt-shear organization. In brief, the best result was obtained for the hybrid particle film having 3-methacryloxypropyltriethoxysilane (MPSEt) as part of the soft shell material. The obtained particle films (Figure 6a,b) clearly exhibited the smallest white border and additionally featured the highest film transparency. The other films containing the alkoxy-silane poly(methacrylates) MPSIsoprop (c) and MPSMeEt (d) featured a significantly larger white powder area as well as a decreased transparency of the inner particle film area.

**Figure 7:**
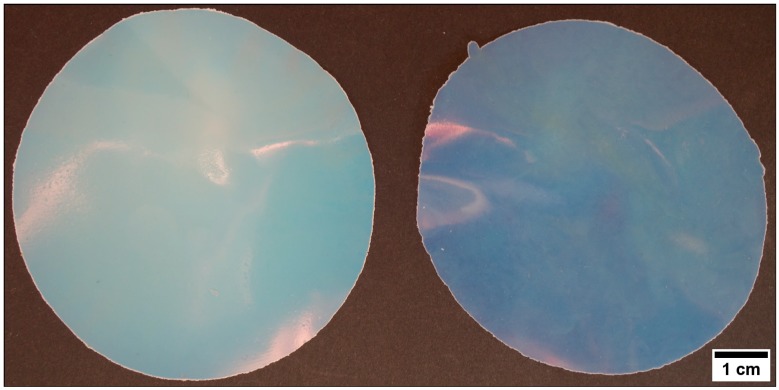
Opal film after melt-shear organization (140 °C, 160 bar, 180 s) of the PS-*co*-PALMA@P(EA-*co*-MPSEt) particles with a particle size of 289 nm (**left**) and 230 nm (**right**).

As further proof for the ability of 3-methacryloxypropyltriethoxysilane-containing (MPSEt) shell particles, these findings were transferred to core-shell particles with P(S-*co*-ALMA) cores having a shell of P(EA-*co*-MPSEt). The resulting hybrid particle films after melt-shear organization are shown in Figure 7.

Compared to the particle films derived from polymethacrylate core-shell particles, PS has higher refractive index (*n*_PS_ = 1.5916) causing a higher net refractive index contrast between the shell and the core material in the processed particle film. Consequently, the obtained hybrid particle films featured a weak iridescent reflection color (Figure 7). As a first result after application of the melt-shear organization technique, the optical impressions clearly pointed out that MPSEt is a suitable monomer for enabling the preparation of a hybrid and meltable shell for PS or PMMA core particles. This approach led to the formation of free-standing hybrid particles films with high transparency (in the case of PMMA as core material) or iridescent reflection color (in the case of PS as core material) after particle processing. The cross-linking reactions of the hybrid particle films after particle processing based on P(S-*co*-ALMA)@P(EA-*co*-MPSEt) will be elucidated in the following section, while morphological aspects and film properties will be investigated by SEM in Section 3.5.

### 3.4. Investigation of the Cross-Linked Siloxane Moieties after Film Formation

**Figure 8:**
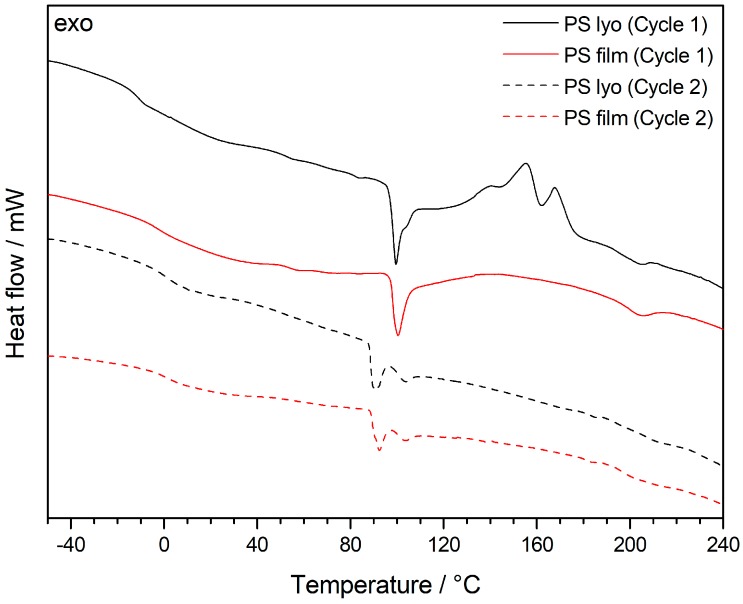
DSC thermograms of P(S-*co*-ALMA)@P(EA-*co*-MPSEt) lyophilized particle powder in comparison with the hybrid particle film after melt-shear organization. DSC measurements were performed with a heating rate of 5 K min^−1^ in nitrogen atmosphere.

For investigation of the cross-linked sites after melt-shear organization of P(S-*co*-ALMA)@P(EA-*co*-MPSEt) particles, DSC measurements were performed from the obtained film samples. Comparison of the corresponding DSC thermograms are given in Figure 8. There, the lyophilized powder of P(S-*co*-ALMA)@P(EA-*co*-MPSEt) and the hybrid particle film after melt-shear organization are shown giving additional insights into the cross-linking state of the hybrid materials.

As explained in Section 3.2, the cross-linking reaction of the lyophilized powder revealed exothermic peaks because of the cross-linking reaction during the DSC measurements in the temperature range between 140 °C and 180 °C. The comparison of the results for the pristine particle powder with the results for the processed particles after melt-shear organization revealed a significant change for the intensity of the exothermic peaks. The decreasing signal intensities for the hybrid film materials compared to the particle material clearly pointed towards a partial in-situ cross-linking during particle processing. However, by comparing the first and second cycle of the DSC measurements, the hybrid particle film after melt-shearing clearly indicated that the siloxane cross-linking reaction was still incomplete after this short time of particle processing (3 min).

**Figure 9:**
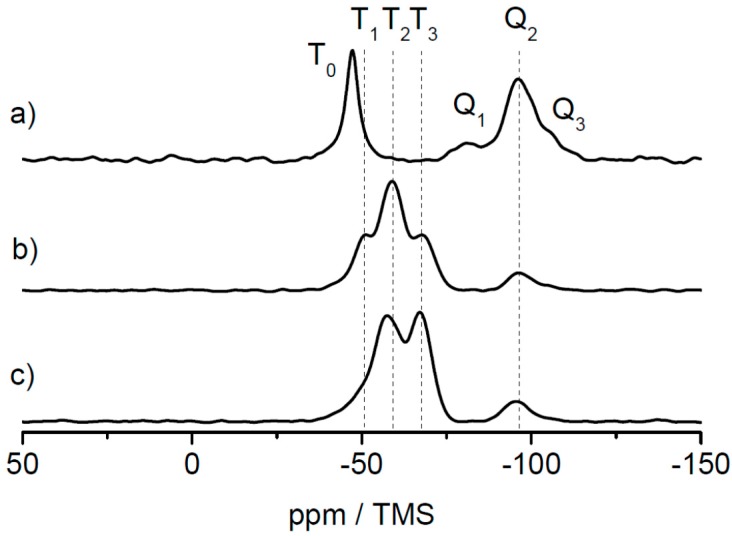
^29^Si CP MAS spectra of samples based on the MPSEt linker system in three different states: (**a**) lyophilized powder, (**b**) hybrid film, and (**c**) hybrid film after heat treatment, and signal assignment of *T_n_* groups (*n* = 0, 1, 2, 3) and *Q_n_* groups (*n* = 1, 2, 3). Spectra were measured at 14 T at a spinning rate of 8 kHz.

As solid-state NMR spectroscopy was the method of choice for investigating the cross-linking capabilities at a molecular level, an additional hybrid film sample was prepared by prolonged thermal treatment at 180 °C for 4 h. The results of the ^29^Si CP MAS measurements for the lyophilized sample, the film sample and the film sample after additional heat treatment are shown in Figure 9 for the samples based on the MPSEt linker system.

As clearly visible in Figure 9, T_0_ groups obtained in the lyophilized sample (Figure 9a) have disappeared in the hybrid film sample (Figure 9b) while T_1_, T_2_, and T_3_ groups have appeared in this sample. This clearly indicates the induced cross-linking via silica during formation of the hybrid film, as described above for the lyophilized samples. Finally, after heat treatment of the hybrid film (Figure 9c), also the signal of the T_1_ group has vanished while the signal of the T_3_ group increased in intensity. This observation is in line with the proceeding cross-linking, which is induced by heat treatment. Similarly, also for the MPSIsoprop- and MPSMeEt-based systems, the hybrid film as well as the hybrid film after heat treatment were investigated by ^29^Si CP MAS NMR, as shown in Figures S11 and S12. Also for both these linker systems, the change of the *T_n_* groups indicates a proceeding of the cross-linking, however this process starts at a higher progress of cross-linking compared to the MPSEt linker system, which is most probably related to different hydrolysis kinetics of the linker molecules.

### 3.5. Morphological Studies on the Hybrid Particle Films

**Figure 10:**
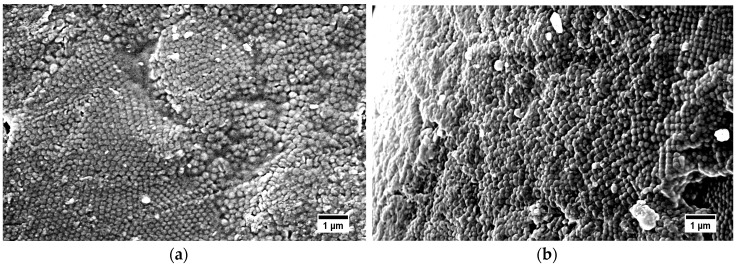
SEM images of the surface (**a**) and cross-section (**b**) of the P(S-*co*-ALMA)@P(EA-*co*-MPSEt) film after processing at 140 °C. The scale bars correspond to 1 µm.

As mentioned in the introduction, core-shell particle processing by using the melt-shear organization enables the possibility to create hybrid particle films without the necessity of a dispersion medium for the particles. As a major drawback of previous investigations for core–shell particle processing by this method, subsequent cross-linking strategies had to be applied after film formation, i.e., either by the application of UV-induced cross-linking chemistry or thermal treatment [23,24,56,57,58,59]. In all cases, cross-linking moieties had to be introduced by extrusion of the core-shell particles mass in order to assure a homogeneous mixing of the cross-linking species. Here, we present a route for avoiding the additional steps by introducing self-cross-linkable siloxane moieties, which enable cross-linking reactions in the last particle processing step based on a novel particle architecture. Finally, the obtained hybrid particle films were investigated by SEM measurements. In Figure 10, the surface and cross-section of obtained hybrid particle films consisting of P(S-*co*-ALMA)@P(EA-*co*-MPSEt) core–shell particles processed at 140 °C are given. Additional SEM images of PMMA-based films with the three different cross-linkers are displayed in Figures S13–S15.

The SEM images of the topography of the hybrid particle film revealed that the material was not ordered throughout the whole material. Some parts of the surface exhibited a sufficient particle order, while some areas featured a significant amount of defects. As a result of the previous sections, particle processing at 140 °C, i.e., in the temperature range of siloxane-cross-linking, led to less ordered particle films. It is assumed that cross-linking reaction competes with particle merging, impeding the preparation of ordered particle films.

**Figure 11:**
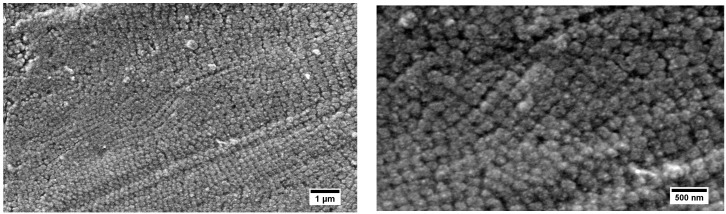
SEM images of the surface from the P(S-*co*-ALMA)@P(EA-*co*-MPSEt) film after processing at 115 °C below the crosslinking temperature.

Therefore, the same core–shell particle material was processed at 115 °C, i.e., significantly below the determined cross-linking reaction temperature for the alkoxy-silane polymethacrylates shell as determined by DSC measurements. For comparison to the particle films processed at 140 °C (Figure 10), the hybrid particle film processed at 115 °C is given in Figure 11. As a result, by processing of the core–shell particles below the identified cross-linking temperature, hybrid core–shell particle films with an increased particle order were obtained. An estimation of the particle to particle distances out of the SEM images given in Figure 10 and Figure 11 revealed a value of about 198 ± 15 nm.

## 4. Conclusions

In the present study, the capability of a novel hybrid cross-linking strategy for the preparation of colloidal crystal films from tailored core-shell particles was examined. The controlled synthesis of core-shell particles was accomplished by starved-feed emulsion polymerization for different alkoxysilane cross-linkers, i.e., 3-methacryloxypropyltriethoxysilane (MPSEt), 3-methacryloxypropyl-triisopropoxysilane (MPSIsoprop), and 3-methacryloxypropyl-tris(methoxyethoxy)silane (MPSMeEt) in the shell of the particle architecture. The hybrid core-shell particles were studied by DLS measurements and TEM, proving the success and excellent control over the polymerization. After lyophilization of the obtained particle dispersions, the particle powder was processed by the melt-shear organization process and colloidal crystal films were obtained in one step. As proven by DSC and MAS NMR experiments, the cross-linking reaction was self-induced during this processing step at around 115 °C. Further thermal treatment increased the crosslinking density within the material. The shell composition of ethyl acrylate and 3-methacryloxypropyltriethoxysilane revealed the optimum results with respect to film formation, processing capabilities, as well as intrinsic order of the underlying particle domains. Finally, the hard core material was varied from PMMA to PS in order to influence the refractive index contrast and hence to gain access to hybrid particle films with iridescent colors in one step. The herein reported insights into self-crosslinking properties and convenient particle order strategy will be extended to functional polymer systems. This will pave the way to applications in the fields of smart stimuli-responsive colloidal crystal films as optical sensors.

## Supplementary Materials

The following are available online at www.mdpi.com/2079-4991/7/11/390/s1, Figures S1–S4: TEM images of the synthesized core-shell particles; Figure S5: DLS measurement of P(S-*co*-ALMA) particles and core-shell particles; Figure S6: DSC measurement of P(MMA-*co*-ALMA)@P(EA-*co*-MPSEt) lyophilized particle powder; Figure S7: Structural possibilities of cross-linking revealed by ^29^Si solid-state NMR; Figures S8–S10: ^13^C static NMR spectra of the neat linkers with signal assignment, and comparison with ^13^C CP MAS spectra of lyophilized powder samples; Figures S11 and S12: ^29^Si CP MAS spectra of MPSIsoprop- and MPSMeEt-based samples; Figures S13–S15: SEM images of the films from the P(MMA-*co*-ALMA) particles with different alkoxysilanes.
