# Supplementary material for: Free-Standing and Self-Crosslinkable Hybrid Films by Core–Shell Particle Design and Processing
Source: Nanomaterials (Basel). 2017 Nov 15;7(11):390. doi: 10.3390/nano7110390 (PMC5707607; doi:10.3390/nano7110390)
Supplement: Supplementary file 1 [file nanomaterials-07-00390-s001.pdf]

Free-Standing and Self-Crosslinkable Hybrid Films by Core-Shell Particle Design and Processing

Steffen Vowinkel [1], Stephen Paul [2], Torsten Gutmann [2] and Markus Gallei [1,*]

Supporting Information

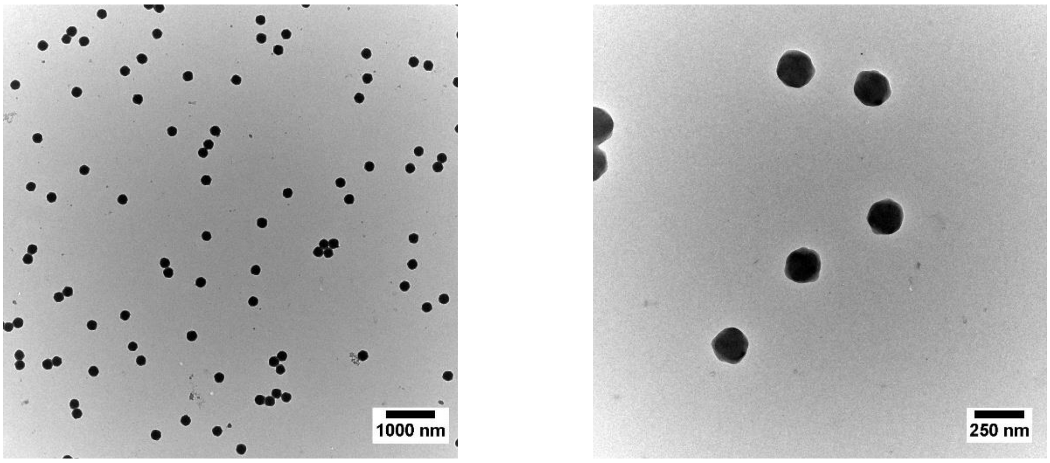

Figure S1. TEM Images of P(MMA-*co*-ALMA)@P(EA-*co*-MPSIsoprop) particles.

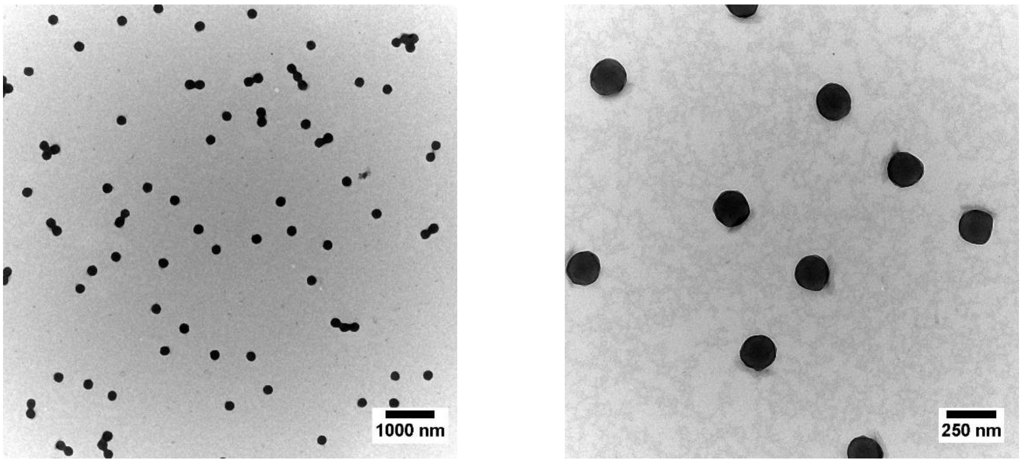

Figure S2. TEM Images of P(MMA-*co*-ALMA)@P(EA-*co*-MPSMeEt) particles.

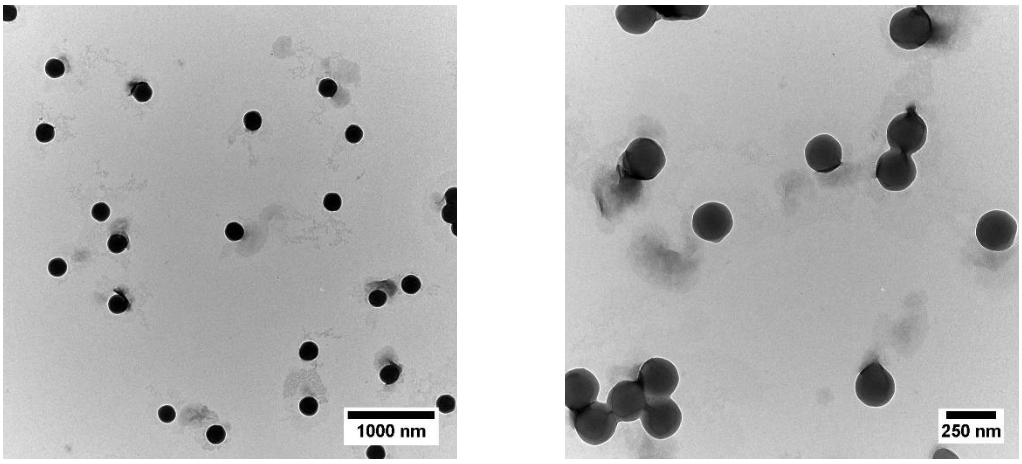

Figure S3. TEM Images of P(S-*co*-ALMA)@P(EA-*co*-MPSEt) particles - 230 nm (DLS).

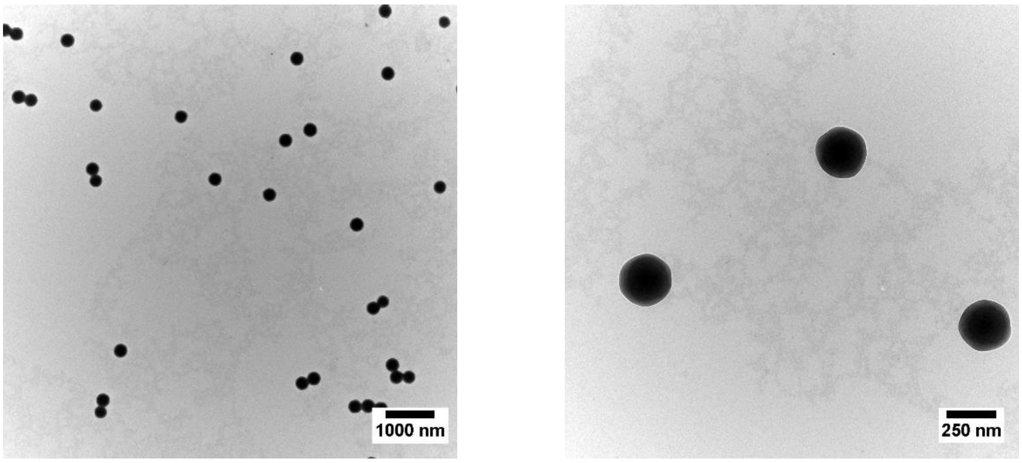

Figure S4. TEM Images of P(S-*co*-ALMA)@P(EA-*co*-MPSEt) particles - 289 nm (DLS).

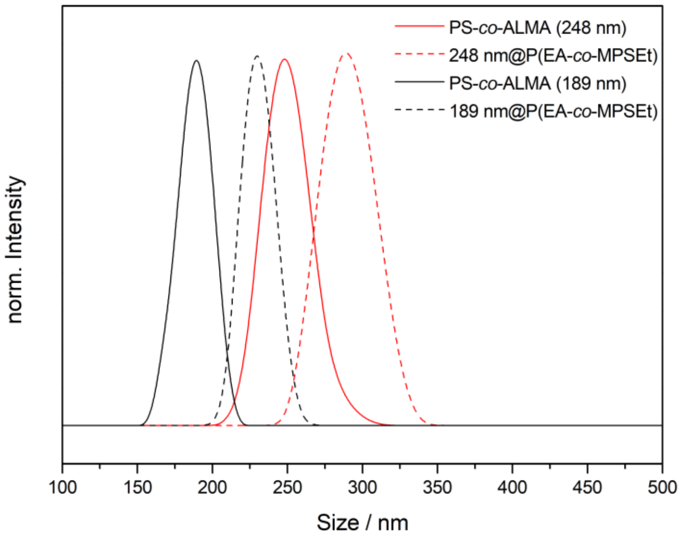

Figure S5. DLS measurement of different sized P(S-*co*-ALMA) particles with corresponding shell of P(EA-*co*-MPSEt).

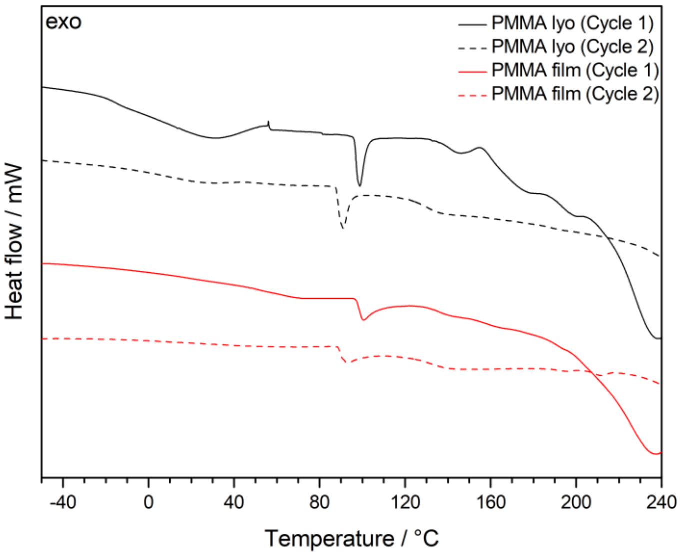

Figure S6. DSC measurement of P(MMA-*co*-ALMA)@P(EA-*co*-MPSEt) lyophilized particle powder in comparison with the film after melt-shear organization with a heating rate of 5 K min^{-1} under nitrogen atmosphere.

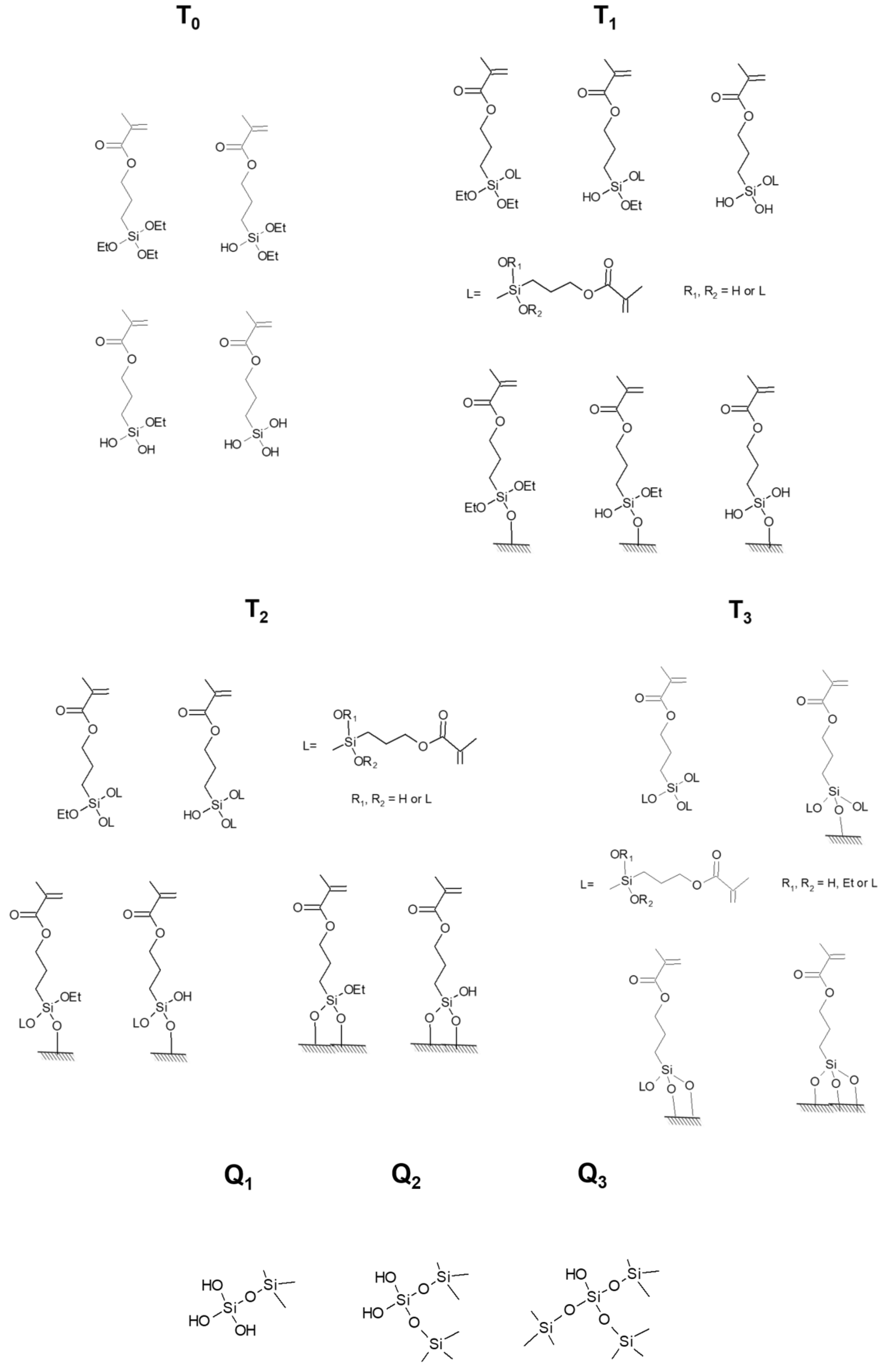

Figure S7. Possibilities of cross-linking of the 3 methacryloxypropyltriethoxysilane (MPSEt) linker indicated by T_n groups (n=0, 1, 2, 3) in the ^{29}Si CP MAS spectra. Note that cross-linking between different linker molecules L is more probable than the binding to bulk silica. The obtained bulk silica indicated by Q_n groups (n= 1, 2, 3) in the ^{29}Si CP MAS spectra is assumed to be formed by decomposition of the organic silane linker.

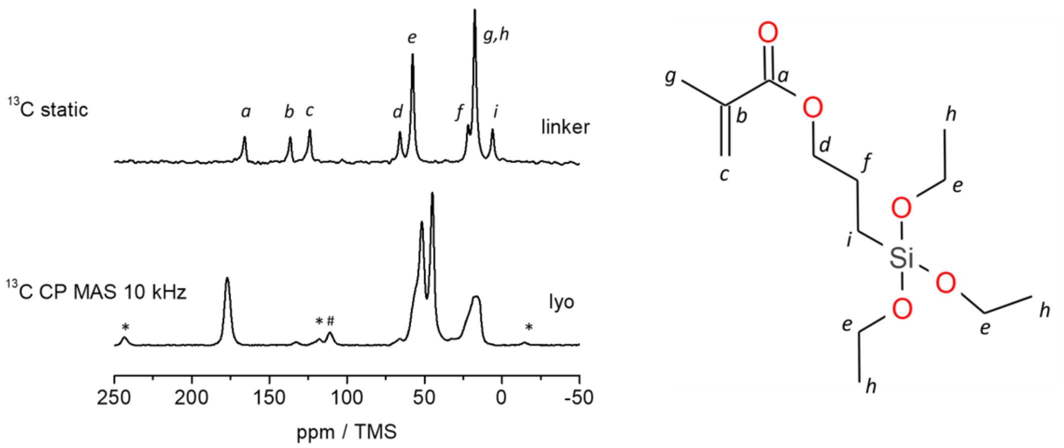

Figure S8. ^{13}C static spectrum of the free linker MPSEt with signal assignments, and corresponding ^{13}C CP MAS spectrum measured at 10 kHz spinning of the lyophilized powder sample prepared with this linker.

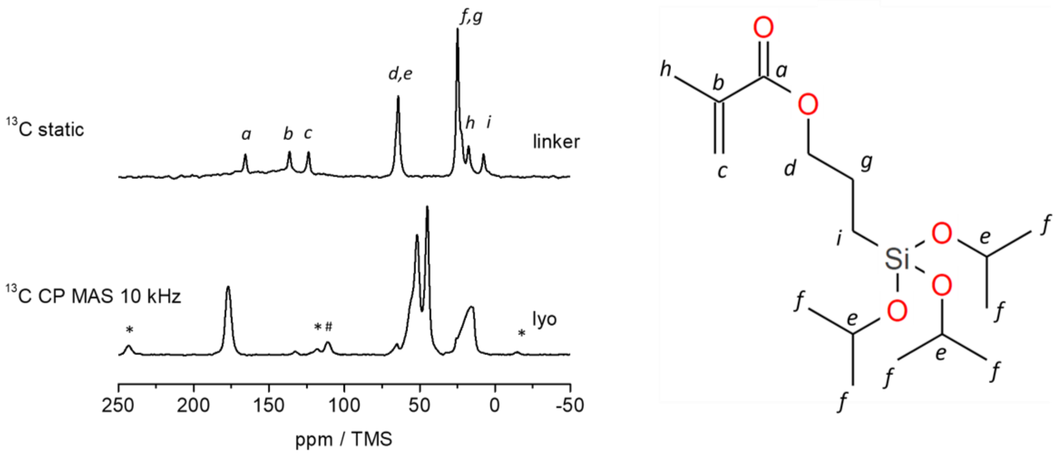

Figure S9. ^{13}C static spectrum of the free linker MPSIsoprop with signal assignments, and corresponding ^{13}C CP MAS spectrum measured at 10 kHz spinning of the lyophilized powder sample prepared with this linker.

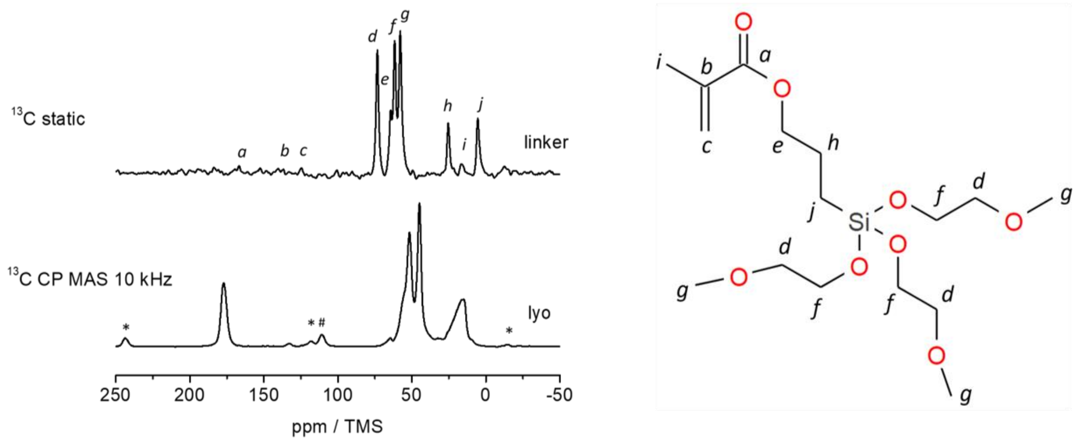

Figure S10. ^{13}C static spectrum of the free linker MPSMeEt with signal assignments, and corresponding ^{13}C CP MAS spectrum measured at 10 kHz spinning of the lyophilized powder sample prepared with this linker.

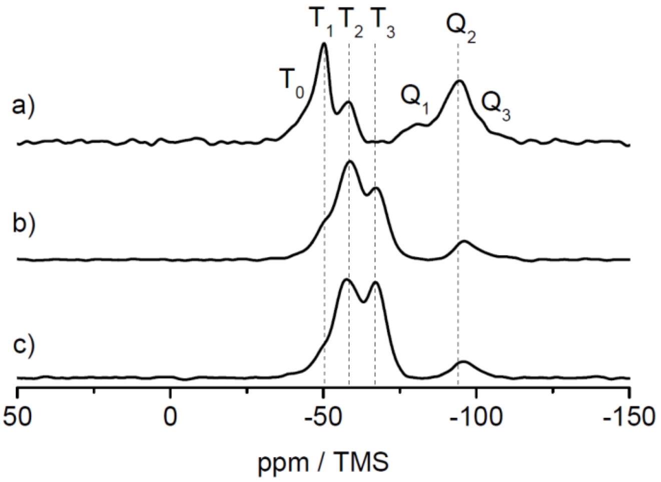

Figure S11. ^{29}Si CP MAS spectra of samples based on the MPSIsoprop linker system in three different states: (a) lyophilized powder, (b) hybrid film and (c) hybrid film after heat treatment, and signal assignment of T_n groups (n=0,1,2,3) and Q_n groups (n=1,2,3). Spectra were measured at 14 T at a spinning rate of 8 kHz.

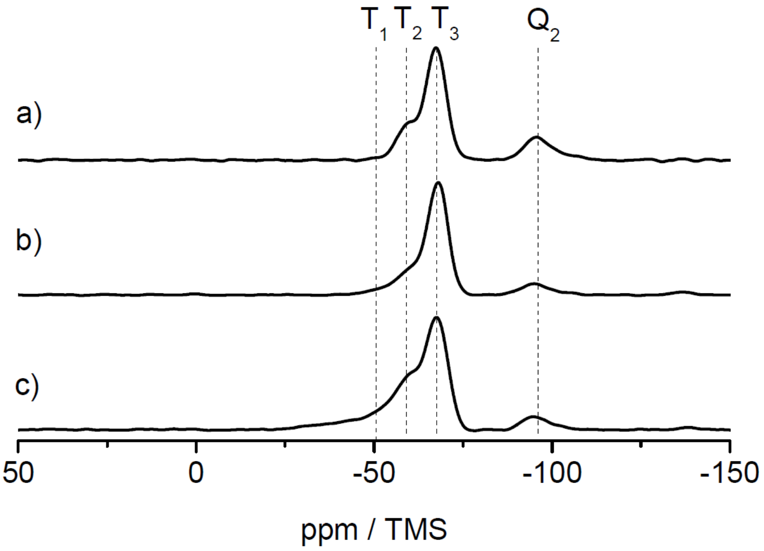

Figure S12. ^{29}Si CP MAS spectra of samples based on the MPSMeEt linker system in three different states: (a) lyophilized powder, (b) hybrid film and (c) hybrid film after heat treatment, and signal assignment of T_n groups (n=0,1,2,3) and Q_n groups (n=1,2,3). Spectra were measured at 14 T at a spinning rate of 8 kHz.

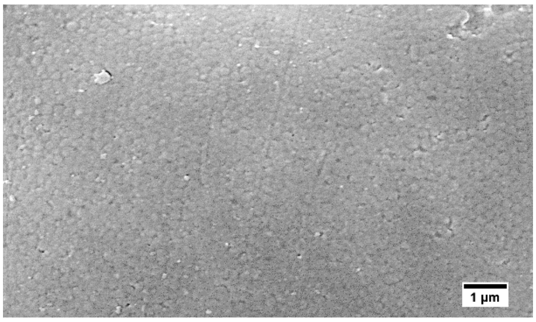

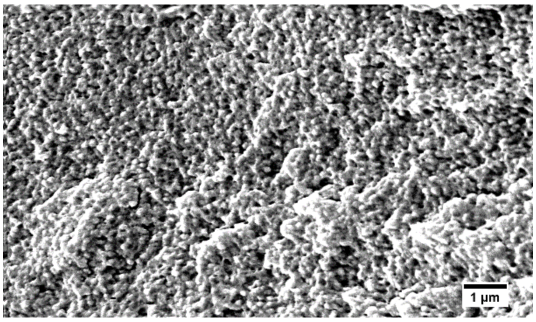

Figure S13. SEM images of the surface (left) and cross-section (right) of the P(MMA-*co*-ALMA)@P(EA-*co*-MPSEt) film after processing at 140°C and 180 bar for 3 minutes.

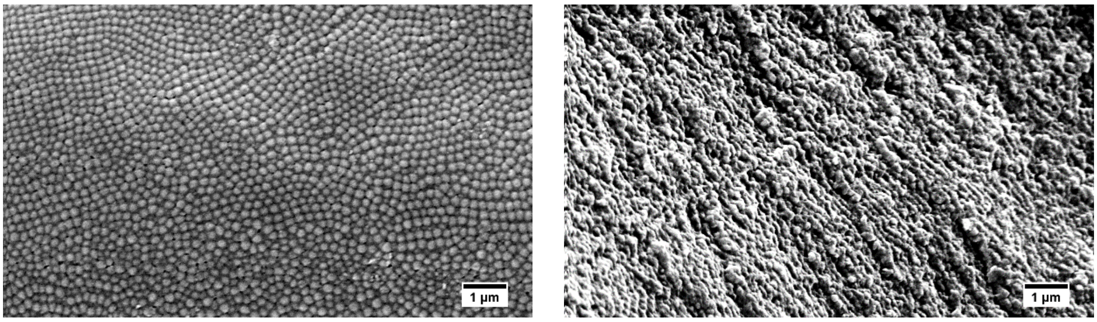

Figure S14. SEM images of the surface (left) and cross-section (right) of the P(MMA-*co*-ALMA)@P(EA-*co*-MPSIsoprop) film after processing at 140 °C and 180 bar for 3 minutes.

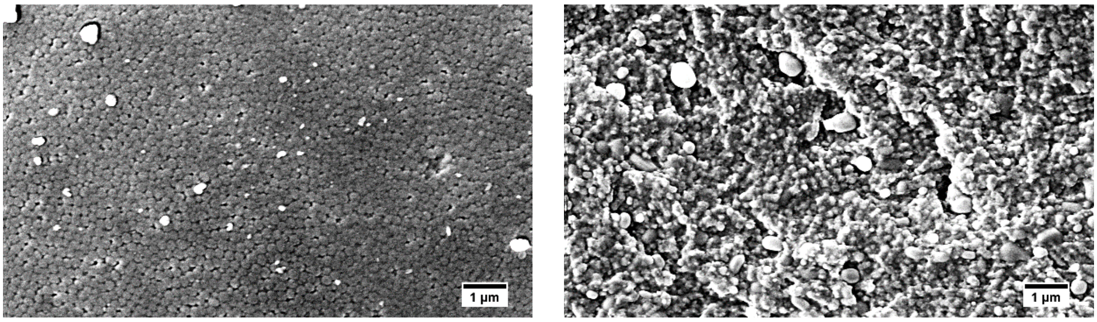

Figure 15. SEM images of the surface (left) and cross-section (right) of the P(MMA-*co*-ALMA)@P(EA-*co*-MPSMeEt) film after processing at 140 °C and 180 bar for 3 minutes.
